# Experimental pain tolerance is associated with dental anxiety– the Tromsø study 2015–2016

**DOI:** 10.1186/s12903-025-06441-0

**Published:** 2025-07-04

**Authors:** Hege Nermo, Natalia Petrenya, Ólöf Anna Steingrímsdóttir, Audun Stubhaug, Christopher Sivert Nielsen, Elin Hadler-Olsen

**Affiliations:** 1 The Public Dental Health Service Competence Center of Northern Norway, Tromsø, Norway; 2 Department of Clinical Dentistry, Faculty of Health Sciences, UiT The Arctic University of Norway, Tromsø, Norway; 3 Department of Physical Health and Ageing, Norwegian Institute of Public Health, Oslo, Norway; 4 Depertment of Research, Oral Health Centre of Expertise in Eastern Norway, Oslo, Norway; 5 Department of Pain Management and Research, Oslo University Hospital, Oslo, Norway; 6 Institute of Clinical Medicine, University of Oslo, Oslo, Norway; 7 Department of Chronic Diseases, Norwegian Institute of Public Health, Oslo, Norway; 8 Department of Medical Biology, Faculty of Health Sciences, UiT The Arctic University of Norway, Tromsø, Norway

**Keywords:** Cross-sectional study, Dental anxiety, Pain sensitivity, Hyperalgesia, Mental health

## Abstract

**Background:**

There are individual differences in pain sensitivity. Dental anxiety may increase the experience of pain during dental treatment, and painful dental treatments may trigger or amplify dental anxiety. This study aimed to explore if experimental pain tolerance in extremities, measured outside the setting of dental treatment, was associated with dental anxiety.

**Methods:**

This cross-sectional study included participants from the seventh survey of the population based Tromsø Study with data on dental anxiety (*n* = 20 186, 40–99 years old, 52.4% women). Dental anxiety was assessed by the Modified Dental Anxiety Scale (MDAS). Tolerance to two experimental pain modalities was evaluated. Pressure pain tolerance (PPT) was measured by computerized cuff pressure on the leg as pressure endured up to a maximum of 100 kPa. Cold-pressor tolerance (CPT) was recorded as time to withdrawal of hand from circulating cold water to a maximum of 120 s. MDAS score across cohort characteristics was assessed with descriptive analyses. The association between pain tolerance and dental anxiety was assessed using binary negative binomial and multinomial logistic regressions adjusted for age, sex, personal finances, smoking, emotional distress, persistent pain, general-, and dental health.

**Results:**

The median MDAS score was 6 (25-percentile: 5, 75-percentile: 9) and the mean was 7.7 (standard deviation: 3.8). Lower experimental pain tolerance was positively associated with MDAS score in fully adjusted models, with p-values for tests of linear trend < 0.001 for both PPT and CPT. Compared to not aborting the test, aborting PPT prior to 48.43 kPa and CPT before 25 s (cut-offs for the lowest tertile among those who aborted the tests) was associated with a 12% and 8% increase in MDAS score, respectively (*p* < 0.001). Multinomial logistic regressions showed that the association between experimental pain tolerance and MDAS scores was most pronounced in the highest MDAS category (≥ 19).

**Conclusions:**

Although causality cannot be interpreted from this cross-sectional study, our findings suggests that a generalized low pain tolerance may render a person more vulnerable for development of dental anxiety. Dentists should have knowledge of and respect individual differences in pain tolerance and strive for optimal pain management in dental settings.

**Supplementary Information:**

The online version contains supplementary material available at 10.1186/s12903-025-06441-0.

## Introduction

Dental anxiety, or the fear of dental treatment, is common in the general population, especially among younger individuals and women [1]. High dental anxiety is often associated with avoidance of dental care, which can have detrimental consequences for the oral health [2]. While the aetiology of dental anxiety is multifactorial [3], pain plays a crucial role in shaping and sustaining it through learned experiences [4, 5]. Additionally, dental anxiety influences how individuals expect, perceive, and remember pain during dental procedures [6]. A longitudinal study of adolescents underscored the link between pain perceptions in dental contexts and dental anxiety, as assessments of pain at the dentist revealed a consistent relationship with variations in dental anxiety levels over time [7].

The experience of pain can be modulated by cognitive factors such as attention, anticipation, catastrophising, and perceived control over pain [8, 9]. Pain sensitivity refers to how a person reacts to a painful stimulus, including their ability to perceive and tolerate pain. There are different ways to assess pain sensitivity, including pain threshold, which is the minimum level of stimulation that provokes pain, and pain tolerance which is the maximum stimulus a person can endure. In experimental settings, pain tolerance can be measured by the time and/or intensity of a painful stimulus an individual can withstand before seeking relief, such as by removing their arm from cold water or releasing a pressure. Demographic, biological, and psychological factors may affect a person’s pain tolerance [10, 11]. Women are often found to be more sensitive to experimental pain than men [12], and younger children have been found to be more sensitive to painful stimuli than older children and adolescents [13]. In adults, pain threshold has been found to increase as we age, whereas pain tolerance seems to remain relatively stable [14]. Pain tolerance is also found to be associated with mental [15, 16] and physical health [17, 18].

While there is compelling evidence that the level of dental anxiety affects a person’s expectations, experiences and recollections of pain from dental treatments [5, 6], there are very few studies assessing if experimental pain tolerance, measured in a setting unrelated to dental treatment, is associated with the level of dental anxiety. The limited number of studies that have been conducted tend to involve a small number of participants and present inconsistent results [19, 20]. However, the only population-based study exploring the association between pain sensitivity and dental anxiety, using pressure pain tolerance as exposure, found a significant association with dental anxiety in a cohort of about 1 700 middle-aged Finnish people [21].

The aim of the present study was to investigate the relationship between experimental pressure- and cold-pressor pain tolerance and dental anxiety. Our hypothesis was that individuals with moderate to high dental anxiety exhibit lower experimental pain tolerance than those with low dental anxiety.

## Materials and methods

### Study design and population

To explore the association between experimental pain tolerance and dental anxiety we used data from the seventh survey of the population based Tromsø Study (Tromsø 7) conducted in 2015–2016 [22]. All inhabitants 40 years or older in Tromsø municipality in Norway were invited, of whom 21 083 (65%) participated. The study was in accordance with the Declaration of Helsinki with informed, written consent from all participants. The Regional Committee for Health Research Ethics approved the study (REK320778). Participants underwent clinical examinations and answered a comprehensive questionnaire assessing sociodemographic, lifestyle as well as somatic-, mental- and oral health-related parameters. An English version of all questionnaire questions used in this study is given in Supplementary file Table S1, which also gives further details on the Tromsø study, and the questions used. This study included participants with complete data on the outcome variable dental anxiety (*n* = 20 186, 95.7% of all participants). Of these, 18 330 (86.9% of all participants) had data on pressure pain tolerance and 17 649 (83.7% of all participants) had data on cold-pressor tolerance, the exposure variables.

### Variables

#### Dental anxiety

The outcome measure in this study was dental anxiety assessed with the Modified Dental Anxiety Scale (MDAS) [23]. MDAS consists of five questions where the respondents rate their emotions to the prospect of a dental visit the day before the visit; in the waiting room just before the visit; in the dental chair about to receive tooth cleaning; tooth drilling; or a local anaesthetic injection in the mouth. There are five ordinal alternatives to each of the questions: (1) “Not anxious”; (2) “Slightly anxious”; (3) “Fairly anxious”; (4) “Very anxious”; and (5) “Extremely anxious”. The marks for each question are summarized giving a score ranging from 5 to 25, where higher scores indicate higher levels of anxiety. The MDAS was analysed both as a continuous and as a categorical outcome. For categorical analysis, the score was divided into four groups (1) 5; (2) 6–10; (3) 11–18; and 4) ≥ 19 to improve clinical interpretability. This grouping reflects increasing severity of dental anxiety, with the uppermost category (MDAS ≥ 19) corresponding to the validated threshold for dental phobia [24].

#### Pain tolerance testing

The exposure variables in this study were pressure- and cold-pressor pain tolerance assessed with the pressure pain tolerance (PPT) test and the cold-pressor tolerance (CPT) test.

#### Pressure pain tolerance (PPT)

PPT was assessed by computerized cuff algometry (NociTech, Aalborg, Denmark) [25] on the calf of the non-dominant leg. A blood-pressure cuff was placed around the calf and inflated by 1 kPa/s up to a maximum safety limit of 100 kPa. The participants were asked to withstand the pain as long as possible and informed to press a button that deflated the cuff if the pain became intolerable. Pain tolerance was defined as the maximum pressure endured if the button was pressed, or right censored if the 100 kPa limit was reached. Participants with open sores, complete lack of sensation in the legs, who were unable to follow instructions, or unwilling to perform the test were excluded. PPT was categorised into (1) Did not abort the test; (2) Aborted the test between 64.27 and 99.49 kPa; (3) Aborted the test between 48.43 and 64.26 kPa; and (4) Aborted the test prior to 48.43 kPa. Cut-offs for categories 2–4 were based on tertiles (group 2 = highest tertile, group 3 = middle tertile, group 4 = lowest tertile of pain tolerance among those who aborted).

#### Cold-pressor tolerance (CPT)

The cold-pressor was a 13-L Plexiglas container with water, where the water temperature was maintained at 3 °C by continuous exchange with a circulating cooling water bath (Julabo PF40-HE, JULABO Labortechnik, GmbH, Germany). The participants submerged the hand and wrist of their non-dominant hand in the water for as long as they could stand, to a maximum of 120 s. Pain tolerance was defined as time to hand withdrawal in seconds if less than 120 s and right censored for participants who endured 120 s. Participants were excluded from the cold-pressor test if they had open sores, were unable to follow instructions (e.g., dementia) or unwilling to take the test. Also, participants with Raynaud syndrome or cold allergy were excluded if they thought that the condition would affect their performance. CPT was categorised into (1) Did not abort the test; (2) Aborted the test after 41–119 s;; (3) Aborted the test after 25–40 s; and (4) Aborted the test before 25 s. Cut-offs for categories 2–4 were based on tertiles (group 2 = highest tertile, group 3 = average tertile, group 4 = lowest tertile of pain tolerance among those who aborted).

### Co-variates

Covariates that have been found to associate with both pain tolerance and dental anxiety were included, namely age [13, 26], sex [1, 12], personal finances [27–29], emotional distress [10, 30], smoking status [31, 32], persistent pain [33, 34], and general- and dental health [17, 29, 34].

Information on age and sex was obtained from the National Population Register. Age was categorised into three groups: (1) 40–49 years; (2) 50–59 years; and (3) 60–99 years. Participants rated their personal finances on a Likert scale from 1) very difficult to 5) very good, which was re-categorised into (1) difficult (original options very difficult and difficult) and (2) average or good (original options average, good and very good). Smoking status was recorded as current, former, or never smoker. Emotional distress was assessed by the 10-item Hopkins Symptom Checklist (HSCL-10) [35] where the respondents rate to what degree they have been bothered by symptoms of anxiety and depression during the last two weeks, with four options (1) not bothered; (2) a little bothered; (3) pretty bothered; and (4) very bothered. Mean item score was calculated and dichotomised into (1) no or low emotional distress (< 1.85) and (2) moderate/high emotional distress (≥ 1.85) [35]. Persistent pain was assessed with the question “Do you have persistent or constantly recurring pain that has lasted for at least 3 months?” with the options yes or no. Participants were also asked to rate their general and dental health with the questions “How do you in general consider your own health to be?” and “How do you consider your own dental health to be?” with options ranging from 1) very good to 5) very poor. Both general health and dental health were re-categorised into (1) good (original options very good and good); (2) average; and (3) poor (original options poor and very poor).

### Statistical analyses

We used the Statistical Package for Social Sciences (SPSS) for Windows version 28 (IMB corporation, Armonk, NY USA) and Stata software (v.18) (StataCorp. 2023. Stata Statistical Software: Release 18. College Station, TX: StataCorp LLC) for statistical analyses. Cronbach’s alpha was used to measure the internal consistency of the MDAS questionnaire. Mean MDAS score with standard deviation (SD) and median MDAS score with 25-pecentile (Q1) and 75-percentile (Q3) was calculated, and differences between groups were analysed with Independent-Samples Kruskal Wallis Test or Independent-Samples Mann-Whitney U test since MDAS was not normally distributed. Correlation between the PPT of the two legs was assessed by intra-class correlation analyses (two-way mixed model, absolute agreement).

The MDAS score did not fulfil the assumptions for performing linear regressions, instead multivariate zero-truncated negative binomial regression was used to assess the association between CPT or PPT and MDAS score, while controlling for putative confounders (sex, age group, personal finances, smoking, emotional distress, chronic pain, general health, and dental health). We present Incidence Rate Ratios (IRR) which represent the change in the MDAS score in terms of percentage increase or decrease, and 95% confidence intervals (CI). PPT and CPT were categorised into tertiles and compared with the category of participants who did not abort the test to ensure simplicity in result interpretation and comparability between PPT and CPT models. Since zero-truncated negative binomial regression may underemphasize clinically meaningful differences, particularly among participants with higher anxiety scores, we also included multinomial logistic regression using the categorized MDAS variable to enable comparison across distinct levels of dental anxiety. We hypothesized that the association between experimental pain tolerance and MDAS score would be stronger among participants with higher anxiety. In the multinomial logistic regression, the group with MDAS = 5 served as a reference category. All covariates included were tested for multicollinearity and none were detected (Tolerance < 1, VIF < 2, and no bivariate correlations > 0.34 (Spearman’s rho)). We assessed for multiplicative interactions between CPT and PPT and each of the covariates by including the cross-product interaction term into the models, and none were detected.

For all analyses p-values < 0.05 were considered statistically significant. Participants with missing data on the included variables were excluded from analyses. The study is reported according to the STROBE statement for cross-sectional studies.

## Results

The Cronbach’s alpha for the MDAS scale was 0.93. The MDAS score ranged from 5 (*n* = 8 435, 41.8%) to 25 (*n* = 125, 0.6%). The median score was 6 (25% percentile: 5; 75% percentile: 9) and the mean score was 7.7 (standard deviation 3.8). Mean and median MDAS score by participant characteristics are shown in Table 1. Women and respondents in the younger age-groups had higher dental anxiety scores than men and older respondents. For all variables included, the MDAS score was different between variable categories, where higher scores were significantly associated with the more unfavourable categories (i.e., having a difficult financial situation, suffering emotional distress or chronic pain, being a smoker, and having poor general or dental health). Furthermore, MDAS score was higher the lower the experimental pain tolerance (Table 1).

**Table 1:** MDAS score by participant characteristics

		MDAS score	
Characteristics	All	Mean (SD)	Median (Q1, Q3)
	20 186	7.7 (3.8)	6.0 (5.0, 9.0)
**Age-group** n (%)			
40–49 years	6 230 (30.9)	8.5 (4.2)	7.0 (5.0, 10.0)
50–59 years	5 821 (28.8)	8.0 (3.9)	7.0 (5.0, 9.0)
60–99 years	8 135 (40.3)	6.8 (3.0)	5.0 (5.0, 8.0)
**Sex** n (%)			
Women	10 574 (52.4)	8.3 (4.1)	7.0 (5.0, 10.0)
Men	9 612 (47.6)	7.1 (3.3)	6.0 (5.0, 8.0)
**Personal finances** n (%)			
Good /average	19 418 (96.5)	7.6 (3.7)	6.0 (5.0, 9.0)
Difficult	703 (3.5)	10.0 (5.5)	8.0 (5.0, 12.0)
**Smoking** n (%)			
Never	8 375 (41.9)	7.4 (3.3)	6.0 (5.0, 9.0)
Former	8 880 (44.4)	7.7 (3.8)	6.0 (5.0, 9.0)
Current	2 754 (13.8)	8.7 (4.8)	7.0 (5.0, 10.0**)**
**Emotional distress** n (%)			
No/little	17 719 (91.3)	7.5 (3.6)	6.0 (5.0, 9.0)
Yes	1 685 (8.7)	9.8 (5.9)	8.0 (6.0, 12.0)
**General health** n (%)			
Good	13 818 (69.0)	7.5 (3.4)	6.0 (5.0, 9.0)
Moderate	5 131 (25.6)	8.1 (4.3)	6.0 (5.0, 10.0)
Poor	1 077 (5.4)	8.9 (4.9)	7.0 (5.0, 11.0)
**Dental health** n (%)			
Good	10 983 (55.4)	7.2 (3.0)	6.0 (5.0, 8.0)
Moderate	6 972 (35.1)	8.0 (3.8)	7.0 (5.0, 10.0)
Poor	1 882 (9.5)	10.1 (5.8)	8.0 (5.0, 13.0)
**Persistent pain** n (%)			
No	11 420 (62.4)	7.4 (3.4)	6.0 (5.0, 9.0)
Yes	6 868 (37.6)	8.1 (4.2)	7.0 (5.0, 10.0)
**Aborted PPT test** n (%)			
No	1485 (8.1)	7.1 (3.1)	6.0 (5.0, 8.0)
64.27–99.49 kPa	5663 (30.9)	7.5 (3.6)	6.0 (5.0, 9.0)
48.43–64.26 kPa	5611 (30.6)	7.7 (3.8)	6.0 (5.0, 9.0)
≤48.42 kPa	5571 (30.4)	7.9 (3.9)	7.0 (5.0, 9.0)
**Aborted CPT test** n (%)			
No	6 711 (38.0)	7.3 (3.4)	6.0 (5.0, 8.0)
41–119 s	3648 (20.7)	7.5 (3.5)	6.0 (5.0, 9.0)
25–40 s	3469 (19.7)	7.9 (3.9)	6.0 (5.0, 9.0)
≤24 s	3821 (21.6)	8.2 (4.1)	7.0 (5.0, 9.0)

Abbreviations: MDAS: Modified Dental Anxiety Scale; PPT: pressure pain tolerance; CPT: cold-pressor tolerance; Q1: 25-percentile; Q3: 75-percentile

Table 1: Differences between categories were assessed with Independent-Samples Kruskal Wallis Test (for variables with two categories) or Independent-Samples Mann-Whitney U Test (more than two categories). *P* < 0.001 for all differences except between never- and former smokers, between the 64.27-99.49 kPa and 48.43-64.26 kPa PPT group (*p* > 0.05) and between the 41–119 s and 25–40 s CPT groups (*p* = 0.02).

To address if participants who underwent the pain test differed from those who did not, we cross-tabulated participants who did and did not undergo the PPT and the CPT tests. This showed that women and participants with moderate or severe symptoms of emotional distress were over-represented among those who did not undergo the tests (*p* < 0.001), while there were no significant differences in level of dental anxiety (*p* = 0.192 PPT test and *p* = 0.308 CPT test). There was no difference in CPT-tolerance between those who underwent the PPT test and those who did not (*p* = 0.958), whereas those who underwent the CPT test had significantly higher PPT tolerance than those who did not undergo the CPT test (*p* < 0.001). Details are shown in Supplementary file Table S2.

In adjusted negative binomial regression models, aborting the PPT test and CPT test was significantly associated with higher MDAS scores (Table 2). Compared to not aborting the test, aborting PPT prior to 48.43 kPa and CPT before 25 s was associated with a 12% and 8% increase in MDAS score, respectively (*p* < 0.001). There was a linear trend between PPT and CPT and MDAS score (*p* < 0.001), with higher IRR the lower the experimental pain tolerance. Supplementary File Figure S1a and b illustrate the linear trend of the expected MDAS score values for PPT test and CPT test categories using predictive margins. Multinomial logistic regression showed that compared to not aborting the test, aborting PPT prior to 48.43 kPa and CPT before 25 s was associated: (1) with a 3.6-fold and 1.6-fold increased relative risk of reporting MDAS score ≥ 19 than reporting MDAS score = 5, respectively; (2) with a 1.9-fold and 1.7-fold increased relative risk of reporting MDAS score 11–18 than reporting MDAS score = 5; respectively; (3) with a 1.9-fold and 1.7-fold increased relative risk of reporting MDAS score 6–10 than reporting MDAS score = 5, respectively (Table 3). Compared to not aborting the test, aborting PPT between 48.43 and 64.26 kPa and CPT between 25 and 40 s was associated: (1) with a 3.0-fold and 1.6-fold increased relative risk of reporting MDAS score ≥ 19 than reporting MDAS score = 5, respectively; (2) with a 1.5-fold and 1.2-fold increased odds of reporting MDAS score 11–18 than reporting MDAS score = 5; respectively; (3) with a 1.3-fold and 1.2-fold increased odds of reporting MDAS score 6–10 than reporting MDAS score = 5, respectively.

**Table 2:** Associations between experimental pain tolerance and MDAS score as a scale variable

MDAS score	IRR (95% CI)*	*p*
Aborted PPT test		
No	Reference	
64.27–99.49 kPa	1.06 (1.03, 1.09)	< 0.001
48.43–64.26 kPa	1.09 (1.06, 1.12)	< 0.001
≤ 48.42 kPa	1.12 (1.09, 1.15)	< 0.001
**P for linear trend < 0.001**		
**Aborted CPT test**		
No	Reference	
41–119 s	1.02 (1.00, 1.04)	< 0.001
25–40 s	1.06 (1.04, 1.08)	< 0.001
≤ 24 s	1.08 (1.06, 1.10)	0.047
**P for linear trend < 0.001**		

* Zero-truncated negative binomial regression adjusted for age, sex, personal finances, smoking, emotional distress, persistent pain, general-, and dental health. Abbreviations: IRR: Incidence Rate Ratio; CI: confidence interval; MDAS: Modified Dental Anxiety Scale; PPT: pressure pain tolerance; CPT: cold pressor tolerance

**Table 3:** Associations between experimental pain tolerance and categories of MDAS score

Aborted pressure pain tolerance test			Aborted cold-pressor tolerance test		
Base outcome MDAS score 5	RRR (95% CI)	P*	Base outcome MDAS score 5	RRR (95% CI)	P*
MDAS score 6–10			MDAS score 6–10		
No	Reference		No	Reference	
64.27–99.49 kPa	1.07 (0.93, 1.21)	0.346	41–119 s	1.11 (1.01, 1.22)	0.031
48.43–64.26 kPa	1.27 (1.11, 1.45)	0.001	25–40 s	1.18 (1.07, 1.31)	0.001
≤ 48.42 kPa	1.36 (1.18, 1.56)	< 0.001	≤ 24 s	1.21 (1.01, 1.22)	0.001
**MDAS score 11–18**			**MDAS score 11–18**		
No	Reference		No	Reference	
64.27–99.49 kPa	1.43 (1.14, 1.79)	0.002	41–119 s	1.17 (1.00, 1.36)	0.050
48.43–64.26 kPa	1.55 (1.23, 1.94)	< 0.001	25–40 s	1.41 (1.21, 1.64)	< 0.001
≤ 48.42 kPa	1.89 (1.50, 2.39)	< 0.001	≤ 24 s	1.71 (1.47, 1.97)	< 0.001
**MDAS score ≥ 19 > 18**			**MDAS score ≥ 19**		
No	Reference		No	Reference	
64.27–99.49 kPa	2.12 (1.21, 3.70)	0.009	41–119 s	1.02 (0.75, 1.38)	0.901
48.43–64.26 kPa	3.00 (1.72, 5.24)	< 0.001	25–40 s	1.57 (1.18, 2.08)	0.002
≤ 48.42 kPa	3.63 (2.07, 6.37)	< 0.001	≤ 24 s	1.59 (1.20, 2.09)	0.001

* Multinomial logistical regression adjusted for age, sex, personal finances, smoking, emotional distress, persistent pain, general-, and dental health. Abbreviations: RRR: Relative Risk Ratio Risk; CI: confidence interval; MDAS: Modified Dental Anxiety Scale

## Discussion

In the current study we have explored the hypothesis that lower experimental pain tolerance is associated with dental anxiety in a large, general adult population cohort. We found that both PPT and CPT were associated with dental anxiety, with stronger associations observed among individuals with higher MDAS scores.

The association between dental anxiety and expectance or experience of pain during dental treatment is well recognised [5]. This association is often explained by the fact that fear increases pain sensitivity and, thereby, the experience of pain [36], suggesting that the fear precedes the pain. However, the sensitivity to pain varies between individuals due to both genetic [10, 37] and acquired or environmental factors [37, 38]. Therefore, a general high pain sensitivity may predispose individuals to dental anxiety. In Tromsø 7, pain tolerance was measured outside the setting of dental procedures, in a controlled, standardized set-up. Nevertheless, the participants brought with them their personality, level of stress and health challenges to the examination, which may have influenced their test results. In our analyses, though, experimental pain tolerance was significantly associated with dental anxiety after adjusting for emotional distress, smoking, self-reported general and dental health, as well as putative life stressors such as persistent pain and personal finances. Thus, our results suggest that a low general pain tolerance may render a person more susceptible to dental anxiety. Given the strong association between experiences of pain in dental settings and the development of dental anxiety [3], our findings are not surprising.

The association between experimental pain tolerance and dental anxiety has, to the best of our knowledge, only been explored in one previous epidemiological study including 1736 46-year-olds in Finland [21]. They also found associations between pressure pain tolerance and dental anxiety. However, a study assessing the associations between dental anxiety, neuroticism, and pain sensitivity in 188 twins [19], found no correlation between pain sensitivity and dental anxiety. The discrepancies between these studies may be due to different ways of measuring pain sensitivity, where the twin study used a VAS-scale rating of pain intensity whereas the present study and the Finnish study used pain tolerance or pain threshold as the measure of pain sensitivity. In the current study, we used two different pain modalities, giving similar results, strengthening the reliability of the association. The association was stronger for PPT than CPT, suggesting that sensitivity to pressure may be more relevant in a dental setting than sensitivity to the cold pressor. Our findings do not contradict that dental fear and psychological factors affect how painful a given dental procedure is perceived but rather add an important aspect to the association between pain experience and dental anxiety. A patient who seems to have relatively low pain tolerance during dental procedures may not necessarily be fearful or anxious but rather have generalized high pain sensitivity and need more careful pain management, such as local anaesthesia, to reduce the risk of developing dental anxiety.

Women generally have lower experimental pain tolerance than men [12, 38] and a higher prevalence of dental anxiety [1]. The mechanisms causing theses sex differences are complex and involve biological factors (e.g., sex hormones [39, 40], psychological factors such as personality traits and emotional processing [41, 42], as well as social factors including the perceived acceptance of expressing anxiety or pain among men and women [38, 43]. Our findings suggest that women’s generally lower pain tolerance may also contribute to the sex difference in prevalence of dental anxiety [38, 44]. 

The results of the current study, combined with the contemporary understanding of pain perception - that it is strongly influenced by expectations and modified through learning [8] - have implications for both the prevention and management of dental anxiety. Together, these findings highlight the importance of systematically assessing patients’ pain expectations and previous experiences in clinical practice to identify those with lower pain tolerance and a higher risk of dental anxiety. Since effective endogenous pain inhibition enhances pain tolerance, educating patients about pain mechanisms and management in the dental context may be crucial in preventing or reducing dental anxiety and improving patient outcomes. This is supported by findings from Van Oosterwijck et al. [45], who demonstrated that pain physiology education can improve health status and endogenous pain inhibition, highlighting the potential benefits of such educational interventions.

### Strengths and weaknesses of the study

The Tromsø7 study is the world’s largest study of experimental pain tolerance. Sampling is population based with a high response rate; thus, the study is reasonably representative of the general middle-aged and old population in the municipality. However, as participation requires meeting on-site in person, seriously ill and nursing home patients are hardly represented. All measurements were performed according to research protocols by trained staff, with objective measurements of pain tolerance. The present study also has some limitations. The study is cross-sectional, thus the associations found may not be causal. Also, the association between pain tolerance and dental anxiety is likely to be bi-directional. This means that individuals with lower pain tolerance might have been more easily conditioned to dental fear. On the other hand, dental fear due to painful dental procedures might have provoked fear of pain in general and pain catastrophizing, which in turn would have further affected the pain experience. We found that women and individuals suffering emotional distress were overrepresented among those that had declined the pain tests in Tromsø 7, whereas no differences were seen in level of dental anxiety between those who underwent the tests and those who did not. Even though we adjusted models for important confounders, some unmeasured confounders could have an impact on the study results, such as personality traits (neuroticism) and affective dimensions of pain (pain-related anxiety).

## Conclusions

Although causality cannot be interpreted from this cross-sectional study, our findings suggest that a generalized low pain tolerance may render an individual more susceptible to developing dental anxiety. The current study adds new perspectives to the association between pain and dental anxiety that may be useful in daily dental practice.

## Electronic supplementary material

Below is the link to the electronic supplementary material.

Supplementary Material 1

## Data Availability

The datasets analyzed are available upon application to Tromsøundersøkelsen: Tromsøundersøkelsen| UiT.

## Abbreviations

MDAS: Modified dental anxiety scale
PPT: Pressure pain tolerance
CPT: Cold–pressor tolerance
SPSS: Statistical package for social sciences
IRR: Incidence Rate Ratio
RRR: Relative Risk Ratio
CI: Confidence interval

## References

[1] Silveira ER, Cademartori MG, Schuch HS, Armfield JA, Demarco FF. Estimated prevalence of dental fear in adults: A systematic review and meta-analysis. J Dent. 2021;108:103632. 33711405 10.1016/j.jdent.2021.103632

[2] Armfield JM. What goes around comes around: revisiting the hypothesized vicious cycle of dental fear and avoidance. Community Dent Oral Epidemiol. 2013;41(3):279–87. 23004917 10.1111/cdoe.12005

[3] Beaton L, Freeman R, Humphris G. Why are people afraid of the dentist? Observations and explanations. Med Princ Pract. 2014;23(4):295–301. 24356305 10.1159/000357223 PMC5586885

[4] de Jongh A, Muris P, ter Horst G, Duyx MP. Acquisition and maintenance of dental anxiety: the role of conditioning experiences and cognitive factors. Behav Res Ther. 1995;33(2):205–10. 7887880 10.1016/0005-7967(94)p4442-w

[5] Lin CS, Wu SY, Yi CA. Association between anxiety and pain in dental treatment: A systematic review and Meta-analysis. J Dent Res. 2017;96(2):153–62. 28106507 10.1177/0022034516678168

[6] Kent G. Memory of dental pain. Pain. 1985;21(2):187–94. 3982842 10.1016/0304-3959(85)90288-X

[7] Nermo H, Willumsen T, Johnsen JK. Changes in dental anxiety among 15- to 21-year-olds. A 2-year longitudinal analysis based on the Tromso study: fit futures. Community Dent Oral Epidemiol. 2019;47(2):127–33. 30408210 10.1111/cdoe.12434

[8] Wiech K. Deconstructing the sensation of pain: the influence of cognitive processes on pain perception. Science. 2016;354(6312):584–7. 27811269 10.1126/science.aaf8934

[9] Wiech K, Tracey I. Pain, decisions, and actions: a motivational perspective. Front Neurosci. 2013;7:46. 23565073 10.3389/fnins.2013.00046 PMC3613600

[10] Patanwala AE, Norwood C, Steiner H, Morrison D, Li M, Walsh K, et al. Psychological and genetic predictors of pain tolerance. Clin Transl Sci. 2019;12(2):189–95. 30468309 10.1111/cts.12605 PMC6440569

[11] Fales JL, Schmaling KB, Culbertson MA. Acute pain sensitivity in individuals with borderline personality disorder: A systematic review and meta-analysis. Clin Psychol Sci Pract. 2021;28(4):341–57.

[12] Bartley EJ, Fillingim RB. Sex differences in pain: a brief review of clinical and experimental findings. Br J Anaesth. 2013;111(1):52–8. 23794645 10.1093/bja/aet127 PMC3690315

[13] El Tumi H, Johnson MI, Dantas PBF, Maynard MJ, Tashani OA. Age-related changes in pain sensitivity in healthy humans: A systematic review with meta-analysis. Eur J Pain. 2017;21(6):955–64. 28230292 10.1002/ejp.1011

[14] Lautenbacher S, Peters JH, Heesen M, Scheel J, Kunz M. Age changes in pain perception: A systematic-review and meta-analysis of age effects on pain and tolerance thresholds. Neurosci Biobehav Rev. 2017;75:104–13. 28159611 10.1016/j.neubiorev.2017.01.039

[15] Adler G, Gattaz WF. Pain perception threshold in major depression. Biol Psychiatry. 1993;34(10):687–9. 8292672 10.1016/0006-3223(93)90041-b

[16] van Middendorp H, Lumley MA, Jacobs JW, Bijlsma JW, Geenen R. The effects of anger and sadness on clinical pain reports and experimentally-induced pain thresholds in women with and without fibromyalgia. Arthritis Care Res (Hoboken). 2010;62(10):1370–6. 20506177 10.1002/acr.20230

[17] Fladseth K, Lindekleiv H, Nielsen C, Ohrn A, Kristensen A, Mannsverk J, et al. Low pain tolerance is associated with coronary angiography, coronary artery disease, and mortality: the Tromso study. J Am Heart Assoc. 2021;10(22):e021291. 34729991 10.1161/JAHA.121.021291 PMC8751909

[18] Myrtveit SM, Skogen JC, Sivertsen B, Steingrimsdottir OA, Stubhaug A, Nielsen CS. Pain and pain tolerance in whiplash-associated disorders: A population-based study. Eur J Pain. 2016;20(6):949–58. 26568528 10.1002/ejp.819 PMC5063105

[19] Vassend O, Roysamb E, Nielsen CS. Dental anxiety in relation to neuroticism and pain sensitivity. A twin study. J Anxiety Disord. 2011;25(2):302–8. 21211939 10.1016/j.janxdis.2010.09.015

[20] Klepac RK, McDonald M, Hauge G, Dowling J. Reactions to pain among subjects high and low in dental fear. J Behav Med. 1980;3(4):373–84. 7194919 10.1007/BF00845291

[21] Kankaanpaa R, Auvinen J, Rantavuori K, Jokelainen J, Karppinen J, Lahti S. Pressure pain sensitivity is associated with dental fear in adults in middle age: findings from the Northern Finland 1966 birth cohort study. Community Dent Oral Epidemiol. 2019;47(3):193–200. 30549076 10.1111/cdoe.12443

[22] Hopstock LA, Grimsgaard S, Johansen H, Kanstad K, Wilsgaard T, Eggen AE. The seventh survey of the Tromso study (Tromso7) 2015–2016: study design, data collection, attendance, and prevalence of risk factors and disease in a multipurpose population-based health survey. Scand J Public Health. 2022;50(7):919–29. 35509230 10.1177/14034948221092294 PMC9578102

[23] Humphris GM, Freeman R, Campbell J, Tuutti H, D’Souza V. Further evidence for the reliability and validity of the modified dental anxiety scale. Int Dent J. 2000;50(6):367–70. 11197195 10.1111/j.1875-595x.2000.tb00570.x

[24] King K, Humphris GM. Evidence to confirm the cut-off for screening dental phobia using the modified dental anxiety scale. Soc Sci Dent. 2010;1(1):21–8.

[25] Jespersen A, Dreyer L, Kendall S, Graven-Nielsen T, Arendt-Nielsen L, Bliddal H, Danneskiold-Samsoe B. Computerized cuff pressure algometry: A new method to assess deep-tissue hypersensitivity in fibromyalgia. Pain. 2007;131(1–2):57–62. 17257757 10.1016/j.pain.2006.12.012

[26] Hagglin C, Berggren U, Hakeberg M, Hallstrom T, Bengtsson C. Variations in dental anxiety among middle-aged and elderly women in sweden: a longitudinal study between 1968 and 1996. J Dent Res. 1999;78(10):1655–61. 10520971 10.1177/00220345990780101101

[27] Poleshuck EL, Green CR. Socioeconomic disadvantage and pain. Pain. 2008;136(3):235–8. 18440703 10.1016/j.pain.2008.04.003 PMC2488390

[28] Miljkovic A, Stipcic A, Bras M, Dordevic V, Brajkovic L, Hayward C, et al. Is experimentally induced pain associated with socioeconomic status? Do poor people hurt more? Med Sci Monit. 2014;20:1232–8. 25029965 10.12659/MSM.890714 PMC4111652

[29] Armfield JM, Spencer AJ, Stewart JF. Dental fear in australia: who’s afraid of the dentist? Aust Dent J. 2006;51(1):78–85. 16669482 10.1111/j.1834-7819.2006.tb00405.x

[30] Halonen H, Nissinen J, Lehtiniemi H, Salo T, Riipinen P, Miettunen J. The association between dental anxiety and psychiatric disorders and symptoms: A systematic review. Clin Pract Epidemiol Ment Health. 2018;14:207–22. 30288171 10.2174/1745017901814010207 PMC6142663

[31] Kallio A, Suominen A, Tolvanen M, Rantavuori K, Jussila H, Karlsson L, et al. Concurrent changes in dental anxiety and smoking in parents of the FinnBrain birth cohort study. Eur J Oral Sci. 2023;131(1):e12912. 36599651 10.1111/eos.12912 PMC10107302

[32] Schistad EI, Stubhaug A, Furberg AS, Engdahl BL, Nielsen CS. C-reactive protein and cold-pressor tolerance in the general population: the Tromso study. Pain. 2017;158(7):1280–8. 28420008 10.1097/j.pain.0000000000000912

[33] Greenspan JD, Slade GD, Bair E, Dubner R, Fillingim RB, Ohrbach R, et al. Pain sensitivity and autonomic factors associated with development of TMD: the OPPERA prospective cohort study. J Pain. 2013;14(12 Suppl):T63–74. e1-6. 24275224 10.1016/j.jpain.2013.06.007 PMC3955992

[34] Beaudette JR, Fritz PC, Sullivan PJ, Ward WE. Oral health, nutritional choices, and dental fear and anxiety. Dent J (Basel). 2017;5(1). 10.3390/dj5010008 PMC5806984 29563414

[35] Strand BH, Dalgard OS, Tambs K, Rognerud M. Measuring the mental health status of the Norwegian population: a comparison of the instruments SCL-25, SCL-10, SCL-5 and MHI-5 (SF-36). Nord J Psychiatry. 2003;57(2):113–8. 12745773 10.1080/08039480310000932

[36] Maggirias J, Locker D. Psychological factors and perceptions of pain associated with dental treatment. Community Dent Oral Epidemiol. 2002;30(2):151–9. 12000356 10.1034/j.1600-0528.2002.300209.x

[37] Nielsen CS, Stubhaug A, Price DD, Vassend O, Czajkowski N, Harris JR. Individual differences in pain sensitivity: genetic and environmental contributions. Pain. 2008;136(1–2):21–9. 17692462 10.1016/j.pain.2007.06.008

[38] Wise EA, Price DD, Myers CD, Heft MW, Robinson ME. Gender role expectations of pain: relationship to experimental pain perception. Pain. 2002;96(3):335–42. 11973007 10.1016/s0304-3959(01)00473-0 PMC2535906

[39] Peyrot C, Brouillard A, Morand-Beaulieu S, Marin M-F. A review on how stress modulates fear conditioning: let’s not forget the role of sex and sex hormones. Behav Res Ther. 2020;129:103615. 32334278 10.1016/j.brat.2020.103615

[40] Bartley EJ, Rhudy JL. Comparing pain sensitivity and the nociceptive flexion reflex threshold across the mid-follicular and late-luteal menstrual phases in healthy women. Clin J Pain. 2013;29(2):154–61. 22688607 10.1097/AJP.0b013e31824c5edb

[41] Whittle S, Yucel M, Yap MB, Allen NB. Sex differences in the neural correlates of emotion: evidence from neuroimaging. Biol Psychol. 2011;87(3):319–33. 21600956 10.1016/j.biopsycho.2011.05.003

[42] Weisberg YJ, Deyoung CG, Hirsh JB. Gender differences in personality across the ten aspects of the big five. Front Psychol. 2011;2:178. 21866227 10.3389/fpsyg.2011.00178 PMC3149680

[43] Garside RB, Klimes-Dougan B. Socialization of discrete negative emotions: gender differences and links with psychological distress. Sex Roles. 2002;47(3):115–28.

[44] Robinson ME, Riley JL 3rd, Myers CD, Papas RK, Wise EA, Waxenberg LB, Fillingim RB. Gender role expectations of pain: relationship to sex differences in pain. J Pain. 2001;2(5):251–7. 14622803 10.1054/jpai.2001.24551

[45] Van Oosterwijck J, Meeus M, Paul L, De Schryver M, Pascal A, Lambrecht L, Nijs J. Pain physiology education improves health status and endogenous pain Inhibition in fibromyalgia: a double-blind randomized controlled trial. Clin J Pain. 2013;29(10):873–82. 23370076 10.1097/AJP.0b013e31827c7a7d

