# Supplementary material for: Experimental pain tolerance is associated with dental anxiety– the Tromsø study 2015–2016
Source: BMC Oral Health. 2025 Jul 4;25:1096. doi: 10.1186/s12903-025-06441-0 (PMC12231863; doi:10.1186/s12903-025-06441-0)
Supplement: Supplementary file 1 — Supplementary Material 1 [file 12903_2025_6441_MOESM1_ESM.docx]

**Supplementary file:**

**Experimental pain tolerance is associated with dental anxiety – the Tromsø Study 2015-2016**

Hege Nermo^1,2^, Natalia Petrenya^1^, Ólöf Anna Steingrímsdóttir^3,4^, Audun Stubhaug^5,6^, Christopher Sivert Nielsen^5,7^, Elin Hadler-Olsen^1,8*^

^1^The Public Dental Health Competence Center of Northern Norway, Tromsø, Norway

^2^Department of Clinical Dentistry, Faculty of Health Sciences, UiT The Arctic University of Norway, Tromsø, Norway

^3^Department of Physical Health and Ageing, Norwegian Institute of Public Health, Oslo, Norway

^4^Depertment of Research, Oral Health Centre of Expertise in Eastern Norway, Oslo, Norway

^5^Department of Pain Management and Research, Oslo University Hospital, Oslo, Norway

^6^Institute of Clinical Medicine, University of Oslo, Oslo, Norway

^7^Department of Chronic Diseases, Norwegian Institute of Public Health, Oslo, Norway

^8^Department of Medical Biology, Faculty of Health Sciences, UiT The Arctic University of Norway, Tromsø, Norway

### Supplementary information about the Tromsø study and questionnaires.

The Tromsø study is a large, population-based health survey that has been repeated 7 times (1). Through clinical examinations and large questionnaires, a broad spectrum of lifestyle and health information is collected from the participants, which is later used in studies covering numerous research questions. There is a team of researchers who decide the questionnaire questions for each study wave. Some questions have been included in all waves to enable longitudinal analyses. The variables generated from the Tromsø study are deposited in a large registry, and researchers apply for variables for specific research project. More information can be found at the Tromsø study’s homepage (2). The questionnaire questions used in this study have been included as co-variates or outcome variables in other studies addressing different research questions.

Of the questions used in this study (Table S1), questions 1-6 have been used and validated in previous rounds of the Tromsø study. Questions 7 is the Modified dental anxiety scale, which is also a well-recognized and validated tool to assess dental anxiety (3).

**Supplementary Tabel S1 Questionnaire questions**

| **#** | **Question** | **Options** | | | | |
| --- | --- | --- | --- | --- | --- | --- |
| 1 | How do you in general consider your health to be? | Excellent | Good | Neither good nor bad | Bad | Very bad |
| 2 | How do you consider your own dental health to be? | 1) Very bad | 2 | 3 | 4 | 5 Excellent |
| 3 | Do you have persistent or constantly recurring pain that has lasted for three months or more? | No | Yes |  |  |  |
| 4 | Do you/did you smoke daily | Never | Yes, now | Yes, previously |  |  |
| 5 | How would you evaluate your finances | Very good | Good | Average | Difficult | Very difficult |
| 6 | Have you experienced some of the following situations during the last week: |  |  |  |  |  |
|  | Sudden fear without apparent reason | No complaint | Little complaint | Pretty much | Very much |  |
|  | Felt afraid or anxious | No complaint | Little complaint | Pretty much | Very much |  |
|  | Faintness or dizziness | No complaint | Little complaint | Pretty much | Very much |  |
|  | Felt tense and upset | No complaint | Little complaint | Pretty much | Very much |  |
|  | Easy blamed yourself | No complaint | Little complaint | Pretty much | Very much |  |
|  | Sleeping problems | No complaint | Little complaint | Pretty much | Very much |  |
|  | Depressed or sad | No complaint | Little complaint | Pretty much | Very much |  |
|  | Felt useless, worthless | No complaint | Little complaint | Pretty much | Very much |  |
|  | Felt that everything is a struggle | No complaint | Little complaint | Pretty much | Very much |  |
|  | Flet hopelessness with regard to future | No complaint | Little complaint | Pretty much | Very much |  |
| 7 | Next are questions on how you experience a visit to the dentist. To what degree would you feel anxious in connection with a visit to the dentist? |  |  |  |  |  |
|  | If you went to your dentist for treatment tomorrow, how would you feel? | Not anxious | Slightly anxious | Fairly anxious | Very anxious | Extremely anxious |
|  | If you were sitting in the waiting room (waiting for treatment), how would you feel? | Not anxious | Slightly anxious | Fairly anxious | Very anxious | Extremely anxious |
|  | If you were about to have a tooth drilled, how would you feel? | Not anxious | Slightly anxious | Fairly anxious | Very anxious | Extremely anxious |
|  | If you were about to have your teeth scaled and polished, how would you feel? | Not anxious | Slightly anxious | Fairly anxious | Very anxious | Extremely anxious |
|  | If you were about to have a local anesthetic injection in your gum, above an upper back tooth, how would you feel? | Not anxious | Slightly anxious | Fairly anxious | Very anxious | Extremely anxious |

### Characteristics of participants who declined the pressure pain tolerance test

Table S4 shows characteristics of participants who declined the pain tolerance tests (pressure pain tolerance (PPT, upper panel) and the cold pressor tolerance (CPT, lower panel)) and those who participated.

**Supplementary Table S2 Characteristics of participants who underwent and declined the pressure pain tolerance (PPT) test and cold pressor tolerance (CPT) test**

|  | **Underwent pressure pain tolerance test** | |  |
| --- | --- | --- | --- |
|  | **Yes** | **No** | **p** |
| **Sex** n (%) |  |  |  |
| Woman | 9 371 (51.1) | 1 203 (64.8) | <0.001^a^ |
| Man | 8 959 (48.9) | 653 (35.2) |  |
| **Dental anxiety**^c^ Median (Q1, Q3) | 6.0 (5.0, 9.0) | 6.0 (5.0, 10.0) | 0.192^b^ |
| **Emotional distress** n (%) |  |  |  |
| No/little | 16 186 (91.6) | 1 533 (88.5) | <0.001^a^ |
| Moderate/severe | 1 486 (8.4) | 199 (11.5) |  |
| **CPT-duration** Median (1Q, 3Q) | 53.0 (27.0, 120.0) | 58.0 (26.0, 120.0) | 0.958^b^ |
| **CPT-aborted test** |  |  |  |
| No | 6 520 (38.1) | 191 (36.9) | 0.607^a^ |
| Yes | 10 612 (61.9) | 326 (63.1) |  |
|  | **Underwent cold pressor tolerance test** | |  |
|  | **Yes** | **No** | **p** |
| **Sex** n (%) |  |  |  |
| Woman | 8 944 (50.7) | 1 630 (64.2) | <0.001^a^ |
| Man | 8 705 (49.3) | 907 (35.8) |  |
| **Dental anxiety**^c^ Median (Q1, Q3) | 6.0 (5.0, 9.0) | 6.0 (5.0, 9.0) | 0.308^b^ |
| **Emotional distress** n (%) |  |  |  |
| No/little | 15 603 (91.6) | 2 116 (89.2) | <0.001^a^ |
| Moderate/severe | 1 428 (8.4) | 257 (10.8) |  |
| **PPT kPa** Median (1Q, 3Q) | 58.6 (46.2, 74.8) | 52.3 (40.7, 67.2) | <0.001^b^ |
| **PPT-aborted test** |  |  |  |
| No | 1 424 (8.3) | 61 (5.1) | <0.001^a^ |
| Yes | 15 708 (91.7) | 1 137 (94.9) |  |

^a^ Pearson Chi-Square test, ^b^ Independent-Samples Mann-Whitney U test ^c^ Assessed with the Modified Dental Anxiety Scale (MDAS). Abbreviations: Q1: 25-percentile; Q3: 75-percentile; CPT: cold-pressor tolerance.

**Supplementary Figure S1 Predictive margins of pain tolerance with 95% confidence intervals**

a)

b)

Supplementary figure S1 shows that aborting the a) pressure pain tolerance test or b) cold pressor tolerance test predicted higher MDAS score with a linear trend (p<0.001)

## References

1. Hopstock LA, Grimsgaard S, Johansen H, Kanstad K, Wilsgaard T, Eggen AE. The seventh survey of the Tromso Study (Tromso7) 2015-2016: study design, data collection, attendance, and prevalence of risk factors and disease in a multipurpose population-based health survey. Scand J Public Health. 2022;50(7):919-29.

2. The seventh survey of the Tromsø Study [Available from: <https://uit.no/research/tromsostudy/project?pid=708909&p_document_id=708030>.

3. Humphris GM, Freeman R, Campbell J, Tuutti H, D'Souza V. Further evidence for the reliability and validity of the Modified Dental Anxiety Scale. Int Dent J. 2000;50(6):367-70.
